# May Fever Trigger Ventricular Fibrillation?

**Published:** 2005-04-01

**Authors:** Jean Luc Pasquié

**Affiliations:** Hôpital Arnaud de Villeneuve, Montpellier, France

**Keywords:** ventricular fibrillation, fever, ventricular ectopy, sudden cardiac death

## Abstract

The clinical precipitants of ventricular fibrillation (VF) remain poorly understood. Clinical factors such as hypoxemia, acidosis or electrolyte imbalance, drug-related toxicity, autonomic nervous system disorders as well as viral myocarditis have been proposed to be associated with sudden cardiac death particularly in patients with structural heart disease. However, In the Brugada syndrome, concurrent febrile illness has been reported to unmask the electrocardiographic features of the Brugada syndrome and be associated with an increased propensity for VF. More recently, a febrile illnesses of infectious etiology was associated to polymorphic ventricular tachycardia or VF in patients with normal hearts and without known repolarization abnormality. In this review we detail this phenomenon and its putative mechanisms.

## Introduction

Ventricular fibrillation (VF) is the main mechanism of sudden cardiac death. Clinical factors such as hypoxemia, acidosis or electrolyte imbalance, drug-related toxicity, autonomic nervous system disorders as well as viral myocarditis have been proposed to be associated with sudden cardiac death particularly in patients with structural heart disease [1]. Emerging evidence implicates triggers dominantly originating from the distal Purkinje arborization and the right ventricular outflow tract (RVOT) in the initiation of VF in a range of clinical conditions [2-7].

Isolated reports in patients with the Brugada syndrome suggest that febrile illnesses may unmask the characteristic electrocardiographic changes that then favour the development of VF [8-15]. However, the clinical precipitants for VF storms in patients with idiopathic VF, without evidence of structural heart disease or known repolarization abnormality, remain poorly characterized. Recently we reported a series of patients in whom a febrile illness precipitated idiopathic VF storm, suggesting that fever may be a precipitant of sudden cardiac death in patients with normal hearts [16].

## Fever and Brugada Syndrome

As summarized in Table 1, isolated case reports have suggested that the phenotypic behaviour of the Brugada syndrome may be influenced by body temperature [8-15]. These reports have observed that not only could the electrocardiographic features of Brugada syndrome manifest but also that it may then be associated with VF during concurrent hyperthermia [8-15]. Such presentations with concurrent febrile illness in the literature have all been males between the ages of 50 and 70 years. The electrocardiographic features of the Brugada syndrome in each case were noted at presentation and gradually returned to normal as fever ceased. However, the Brugada electrocardiographic pattern was not reproducible by pharmacologic testing in all patients (Table 1). Very recently, ajmaline testing was reported to be a valuable test in diagnosis of SCN5A carriers with sensitivity, specificity and positive and negative predictive values of 80, 94, 93 and 83 % respectively [16]. However, considering risk stratification for identifying high-risk patients in Brugada syndrome, SCN5A mutation, VF inducibility by EP study and pharmacologic challenge were not predictive factors of life-threatening arrhythmic events [17].

Morita et al described a 69 year old patient with prior unexplained resuscitated sudden death (5 years ago), who presented with ST segment elevation, T wave alternans and premature ventricular contractions in association with a respiratory infection and mild fever (37.5°C) [10]. In two more patients, one with known Brugada syndrome, a febrile illness was associated to syncope due to polymorphic ventricular tachycardias or VF [8,15]. Dinckal and al reported the dramatic case of a 55 year old male who experienced fever due to pneumonia which unmasked typical ST changes of Brugada syndrome and lead to VF refractory VF storm resulting in the patient’s death [14]. Of particular interest, Mok and colleagues reported the case of a patient whose Brugada ECG pattern was unmasked by hyperthermia secondary to acute cholangitis [12]. In order to explain this phenomenon, they have demonstrated by genetic screening a novel mutation in the SCN5A gene related to a single amino acid substitution (H681P) confirming the diagnosis of Brugada syndrome. This mutation was associated with the loss of transmembrane current and induced a pathologic phenotype that may be elicited by hyperthermia [12]. In vitro expression of this amino acid substitution revealed a shift in both steady-state activation and inactivation of the sodium channel resulting in a dramatic reduction of sodium window current [12]. This temperature-dependency of ionic mechanisms underlying Brugada syndrome have also been described in experimental studies [18]. They hypothetized that at physiological temperature ranges the gating of the sodium channel may be changed by the SCN5A mutation such that the outward current is dramatically increased in the early phases of the right ventricular action potential [18]. Using the patch-clamp technique to study the currents at 32° C they tested a missense mutation (Thr1620Met) in SCN5A in a mammalian cell line and demonstrated a faster decay and slower reactivation of the inward sodium current with a outward shift of the current flowing during phase I. This phenomenon may be responsible for a transmural voltage gradient that may cause the repolarization abnormalities and be responsible for phase 2 reentry [18]. This work favours the hypothesis that some mutations may exhibit temperature-dependency properties and may explain that some patients may be at particularly high risk during febrile illnesses [18,19].

## Fever and Idiopathic Ventricular Fibrillation

In contrast, a paucity of reports (Table 1) describes association of fever and ventricular fibrillation in patients with idiopathic VF. Unlike other polymorphic ventricular tachyarrhythmias, idiopathic VF is generally not related to stress [20], anger or physical activity [21] and spontaneous VF in these patients is initiated by premature ventricular complexes with very short coupling intervals [22].

**Figure 1:**
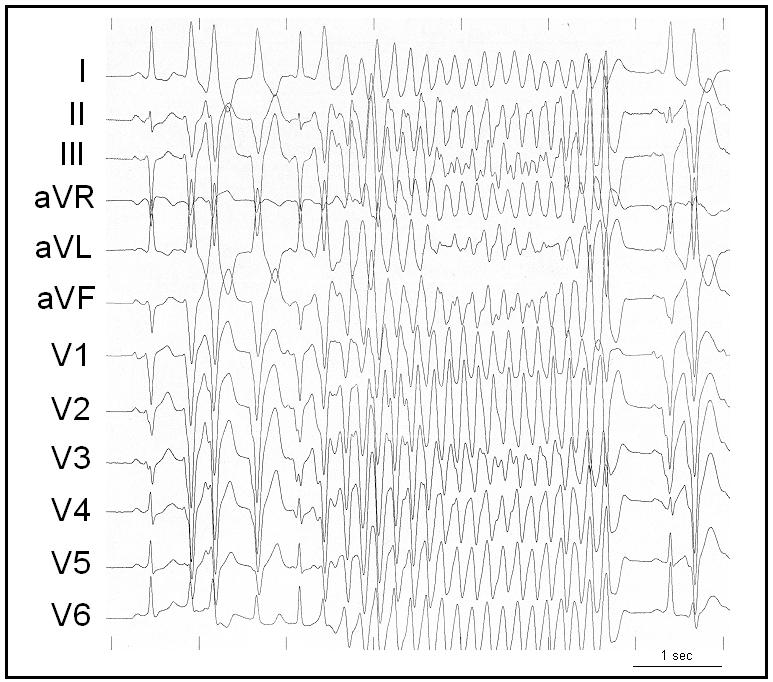
Polymorphic ventricular tachycardia initiated by short coupling ectopy in a 58 year old patient with febrile urinary sepsis and repetitive syncope. Further monitoring of this patient exhibited syncope related to ventricular fibrillation necessitating cardioversion

**Figure 2:**
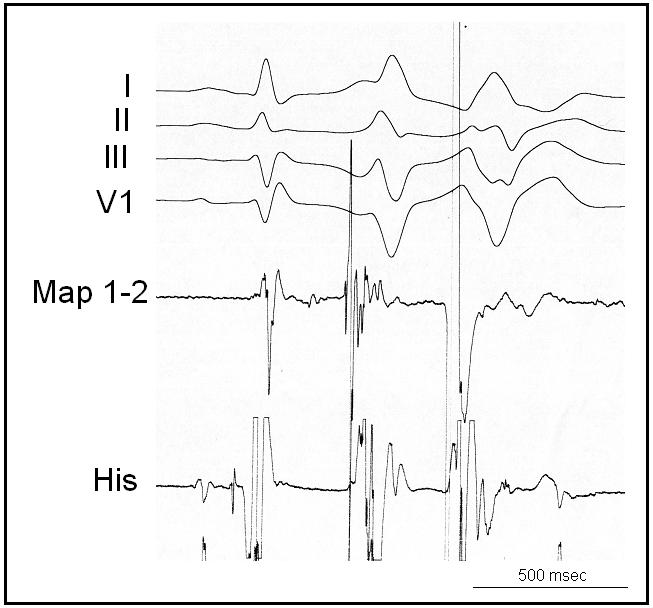
Sharp Purkinje potential preceding the initiating ventricular ectopy in the same patient.

In patients with structural heart disease, the clinical factors that may be associated with ventricular fibrillation are transient ischemia and reperfusion, systemic factors as hypoxemia, acidosis or electrolyte imbalance, neurophysiologic interactions or toxic effects [1]. In patients with normal hearts, viral myocarditis is generally considered one of the main mechanism of sudden cardiac death associated with a febrile illness; although this has been poorly documented and could only be demonstrated by biopsy. We recently reported 3 patients who had no demonstrable structural heart disease or repolarization abnormalities and who presented with episodes of VF storm during a peak of a febrile illness usually occurring at night [23]. They demonstrated short coupled ventricular ectopies (Figure 1). Ectopy morphology was highly suggestive of left Purkinje origin in one, and of muscular origin from RVOT in the other one. In the third patient with recurrent episodes of VF, mapping and ablation was performed confirming the origin of the triggering ectopy from the right Purkinje system (Figure 2). None of our patients had evidence to suggest a viral myocarditis with biological screens, troponin values and viral screens being normal. In addition, one of these patients had a documented bacterial urinary sepsis. A potential role for the autonomic nervous system cannot be excluded as VF occurred at night; however, none of our patients demonstrated a change in repolarization prior to VF storm.

The distal Purkinje arborization has been implicated as the site of origin of triggers initiating VF in a variety of clinical situations. These include patients with VF associated with structural heart disease such as following myocardial infarction [7] abnormal repolarization syndromes [4] and also those with idiopathic VF [2,3]. The Purkinje system while being capable of sustaining spontaneous activity by automaticity, reentry or triggered activity, is known to be sensitive to a variety of clinical situations. The similarity of these observations with those made in the Brugada syndrome, indicates a potentially greater role of concurrent illnesses in the initiation of VF. Indeed, heterogeneous changes in ion-channel expression have been described in Purkinje fibers and ventricular muscle in experimental studies [24] Taken in conjunction with our findings [23], it indicates a potentially greater role of such concurrent illnesses in the initiation of VF. The morphologic features of the short coupled ectopy in the three febrile patients with normal hearts were identical to those previously demonstrated in patients with idiopathic VF [22]. The similar origin of triggers in this series suggests that the current observations may have resulted from increased frequency or malignancy of the triggers by the febrile illness in these patients with idiopathic VF. This series provides new information on the precipitating role of concurrent febrile illness in the initiation of VF in patients with normal hearts. A possible explanation is that some patients may exhibit temperature-dependent modifications of ion channel properties or expression that may facilitate spontaneous activity within Purkinje or RVOT as a potential mechanism for these observations.

## Conclusion

Febrile illness is a common clinical situation managed by a variety of physicians. From the data available so far, it appears that an apparently benign febrile illness may be associated with malignant ventricular arrhythmias in patients with Brugada syndrome and sometimes in the absence of cardiac disease or other factors known to precipitate sudden cardiac death. The mechanism is still unclear but may be related to temperature-dependent modifications of ion channel properties or expression that facilitate spontaneous activity within Purkinje or RVOT as triggers of ventricular fibrillation. We believe that physicians should be aware of this possible phenomenon in all cases of febrile illness associated with syncope.

## Figures and Tables

**Table 1:**
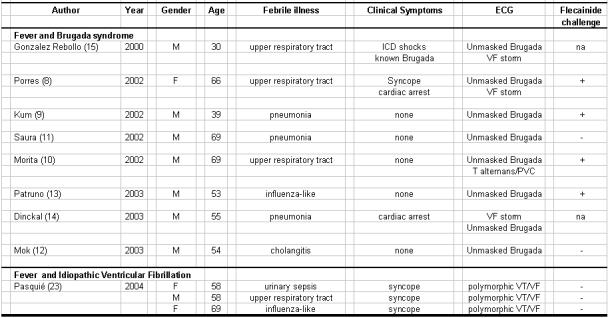
Reports of Fever in patients with Brugada syndrome or Idiopathic ventricular fibrillation
